# Safety and feasibility of umbilical cord blood transplantation in children with neuronal ceroid lipofuscinosis: a retrospective study

**DOI:** 10.1093/stcltm/szae080

**Published:** 2024-10-29

**Authors:** Andrea Bauchat, Veronika Polishchuk, Vanessa A Fabrizio, Jennifer E Brondon, Kristin M Page, Timothy A Driscoll, Paul L Martin, Kris M Mahadeo, Joanne Kurtzberg, Vinod K Prasad

**Affiliations:** Division of Pediatric Transplant and Cellular Therapy, Duke University, 2400 Pratt Street, Box 102502, Durham, NC 27705, United States; Division of Pediatric Hematology, Oncology, and Stem Cell Transplant, Ann & Robert H. Lurie Children’s Hospital of Chicago, 225 E Chicago Ave, Chicago, IL 60611, United States; Colorado Children’s Hospital, Anschutz Medical Campus, University of Colorado, 13123 E 16th Ave, Aurora, CO 80045, United States; Fortrea Inc., 8 Moore Dr, Durham, NC 27703, United States; Department of Pediatrics and Medicine, Medical College of Wisconsin, 8915 W Connell Ct, Milwaukee, WI 53226, United States; Division of Pediatric Transplant and Cellular Therapy, Duke University, 2400 Pratt Street, Box 102502, Durham, NC 27705, United States; Division of Pediatric Transplant and Cellular Therapy, Duke University, 2400 Pratt Street, Box 102502, Durham, NC 27705, United States; Division of Pediatric Transplant and Cellular Therapy, Duke University, 2400 Pratt Street, Box 102502, Durham, NC 27705, United States; Division of Pediatric Transplant and Cellular Therapy, Duke University, 2400 Pratt Street, Box 102502, Durham, NC 27705, United States; Division of Pediatric Transplant and Cellular Therapy, Duke University, 2400 Pratt Street, Box 102502, Durham, NC 27705, United States

**Keywords:** umbilical cord blood, stem cell transplantation, hematopoietic stem cell transplantation, neuronal ceroid lipofuscinoses, batten disease, umbilical cord blood transplantation

## Abstract

Ceroid lipofuscinosis neuronal (CLN) encompasses rare inherited neurodegenerative disorders that present in childhood with clinical features including epilepsy, psychomotor delay, progressive vision loss, and premature death. Published experience utilizing umbilical cord blood transplant (UCBT) for these disorders is limited. This retrospective analysis includes patients with CLN (2, 3, and 5) who underwent UCBT from 2012 to 2020. All subjects (*n* = 8) received standard-of-care myeloablative conditioning. Four also enrolled in clinical trial NCT02254863 and received intrathecal DUOC-01 cells posttransplant. Median age at UCBT was 5.9 years. All subjects achieved neutrophil engraftment with >95% donor chimerism at a median of 28.5 days. Sinusoidal obstructive syndrome was not observed. Severe acute graft-versus-host disease occurred in 12.5%. Other complications included autoimmune hemolytic anemia (25%) and viral reactivation/infection (62.5%). No transplant-related mortality was observed. Two CLN2 patients died, 1 from progressive disease and 1 from unknown cause at days +362 and +937, respectively. With median follow-up of 8 years, overall survival at 100 days and 24 months was 100% and 88%, respectively. Three of 4 CLN3 subjects stabilized Hamburg motor and language scores. While UCBT appears safe and feasible in these patients, given the variable expression and natural history, extended follow-up and further studies are needed to elucidate the potential impact of UCBT on clinical outcomes.

## Graphical Abstract

**Graphical Abstract:**
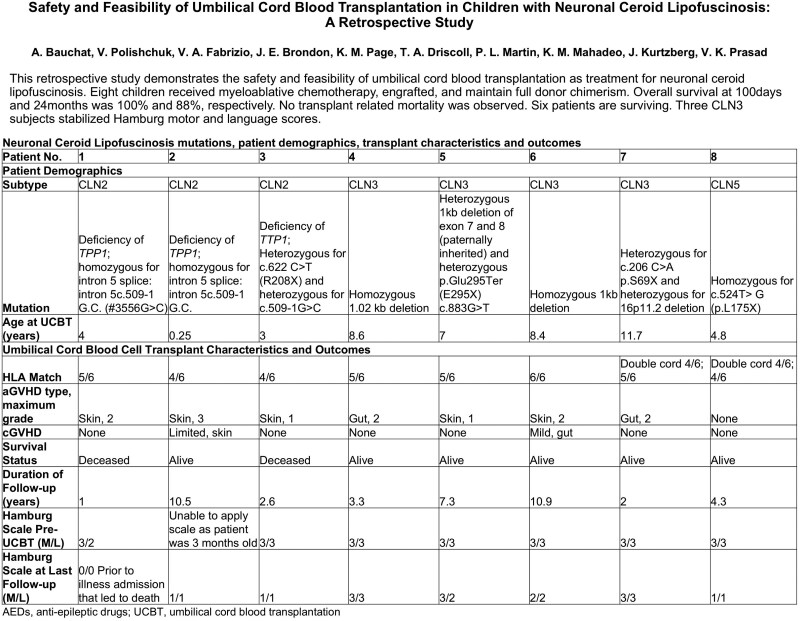

**Lessons learned**

- Umbilical cord blood transplantation is safe and feasible in patients with neuronal ceroid lipofuscinoses demonstrated by successful engraftment, full donor chimerism, and no transplant-related mortality.
- Results suggest potential for stabilization observed in CLN3 patients, although long-term studies are needed to delineate this from the more indolent natural history of this disease subset.
- Further studies are needed to expand the sample size and extend follow-up to determine the full potential benefits of umbilical cord blood transplant on clinical outcomes in neuronal ceroid lipofuscinoses.

## Introduction

Neuronal ceroid lipofuscinoses (NCLs), including Batten disease (BD), are a rare group of inherited lysosomal storage diseases caused by mutations in ceroid lipofuscinosis neuronal (CLN) genes, leading to progressive neurodegeneration from absent lysosomal enzyme activity. Over 400 disease-causing NCL mutations have been reported from over 1200 patients/families, with attempts to characterize genotype/phenotype correlations.^1^ To date, over a dozen autosomal recessive NCL gene variants have been described in childhood and 1 autosomal dominant gene related to the adult form. NCL gene variants involve defective proteins (corresponding genes designated *CLN* for “ceroid lipofuscinosis, neuronal”) and include secreted lysosomal proteins (CLN1/PPT1, CLN2/TPP1, CLN5, CLN10/CTSD, CLN13/CTSF, and CLN11/GRN) or membrane proteins (CLN3, CLN7/MFSD8, and CLN12/ATP13A2), as well as membrane proteins of the endoplasmic reticulum (CLN6 and CLN8), and cytoplasmic proteins (CLN4/DNAJC5 and CLN14/KCTD7), which peripherally associate with cell membranes.^2^ While allelic heterogeneity exists in the *CLN2* gene, 2 mutations (c.5091G→A [splicing error] and c.622C→T [nonsense mutation]) have been identified in more than half of patients with CLN2 disease.^3^ Over 40 and 58 pathogenic variants for CLN3 and CLN5 genes have been reported, respectively. All mutation details can be found in the publicly available NCL mutation database: www.ucl.ac.uk/ncl-disease (accessed on June 15, 2024).

Neuronal ceroid lipofuscinoses are the most prevalent childhood neurodegenerative disorders with the late infantile form predominating (80%).^4^ A tripeptidyl peptidase enzyme replacement, cerliponase alfa, administered intraventricularly, may slow gross motor deterioration in symptomatic patients with late infantile CLN2, or tripeptidyl peptidase 1 (TPP1) deficiency, but is not curative.^5^ Unrelated donor umbilical cord blood transplantation (UCBT) has effectively treated inherited metabolic disorders (IMD) with peroxisomal and lysosomal storage defects by normal enzyme excretion from donor cells cross-correcting deficient cells/tissues and donor-derived glial cell growth in the central nervous system (CNS) specifically advantageous for neurocognitive outcomes.^6^

Nickel et al. described CLN2 disease’s natural history, where patients presented around ages 2-4 years, experienced rapid functional decline and died within their first decade.^3^ The median time between symptom onset and death was 7-8 years. CLN3 disease progression appears slower, spanning over 2 decades. Hamburg Motor and Language (HML) scores may therefore plateau (lower scores reflect advanced disease) over long periods despite progressive brain atrophy with MRI volume loss varying 2.4%-4.7% annually.^7^ CLN5 disease presents between ages 4-7 years with intellectual disability, ataxia, and myoclonic epilepsy.^8^ Behavioral problems at onset are common with CLN3, CLN5, and occasionally atypical CLN2 diseases.^1^ Reported cardiac involvement features repolarization disturbances, ventricular hypertrophy, and sinus node dysfunction.^8^ Video-EEG monitoring may identify choreoathetosis, dystonia, or mixed movement disorders.^8^ Spasticity develops in later stages complicated by spinal scoliosis and joint contractures.^8^ We describe safety and feasibility of utilizing UCBT for BD in patients with CLN2, CLN3, and CLN5.

## Methods

In this retrospective study, all patients with CLN2, CLN3, and CLN5 who underwent HCT from 2012 to 2020 at Duke University were included. Patients received myeloablative conditioning with busulfan, cyclophosphamide, and equine anti-thymocyte globulin.^6^ Graft-versus-host disease (GVHD) prophylaxis consisted of cyclosporine and mycophenolate mofetil and supportive care as previously described.^6^ All patients received seizure prophylaxis during busulfan administration and 6 continued prophylaxis until calcineurin inhibitor therapy completion, progression to requiring anti-epileptic treatment (AED), or as continuation of pre-UCBT AEDs. Five patients enrolled in an IRB-approved phase 1 study (NCT02254863) and received an adjuvant cellular therapy of umbilical cord blood-derived oligodendrocyte-like cells (DUOC-01) intrathecally 28-42 days after UCBT.^9^ This therapy is intended to administer DUOC-01 directly into the CNS, thereby expediting engraftment of donor-derived microglial cells to mitigate progression of neurodegeneration.^9^ Transplant variables were standardly defined.^10^ HML-assessed motor-language domains.^11^ Standard descriptive statistical analysis was performed.^10^

## Results

### UCBT clinical course

Patients were diagnosed with CLN2 (*n* = 3), CLN3 (*n* = 4), or CLN5 (*n* = 1) at a median age of 5.25 years. The median time from diagnosis to UCBT was 246 days and the median age at UCBT was 5.9 years (Table 1). Three patients (CLN2 = 2; CLN5 = 1) diagnosed early due to abnormal genetic testing of an older sibling, were asymptomatic prior to UCBT. Five symptomatic patients had vision loss, seizures, behavioral issues, echolalia/speech deficits, and/or motor deficits at the time of UCBT. Behavioral concerns included erratic and unpredictable actions as well as disruptive and defiant conduct (CNL3 = 2).

**Table 1.** Neuronal ceroid lipofuscinosis mutations, patient demographics, transplant characteristics, and outcomes.

Patient no.	1	2^a^	3^a^	4	5^a^	6	7^a^	8^a^
Patient demographics								
Subtype	CLN2	CLN2	CLN2	CLN3	CLN3	CLN3	CLN3	CLN5
Mutation	Deficiency of *TPP1*; homozygous for intron 5 splice: intron 5c.509-1 G.C. (#3556G>C)	Deficiency of *TPP1*; homozygous for intron 5 splice: intron 5c.509-1 G.C.	Deficiency of *TTP1*; Heterozygous for c.622 C>T (R208X) and heterozygous for c.509-1G>C	Homozygous 1.02 kb deletion	Heterozygous 1kb deletion of exon 7 and 8 (paternally inherited) and heterozygous p.Glu295Ter (E295X) c.883G>T	Homozygous 1 kb deletion	Heterozygous for c.206 C>A p.S69X and heterozygous for 16p11.2 deletion	Homozygous for c.524T>G (p.L175X)
Age at UCBT (years)	4	0.25	3	8.6	7	8.4	11.7	4.8
Sex (male/female)	M	F	F	M	F	M	F	M
Presentation	Severe speech and motor deterioration; seizures; hearing loss	Asymptomatic	Asymptomatic	Blind; seizures; behavioral issues	Visual loss	Blind; staring spells; echolalia	Progressive vision loss (retinal dystrophy with maculopathy); behavior issues; articulatory speech difficulties	Asymptomatic
Lansky Performance	90	100	90	90	100	100	80	100
Umbilical cord blood cell transplant characteristics and outcomes								
HLA match	5/6	4/6	4/6	5/6	5/6	6/6	Double cord 4/6; 5/6	Double cord 4/6; 4/6
aGVHD type, maximum grade	Skin, 2	Skin, 3	Skin, 1	Gut, 2	Skin, 1	Skin, 2	Gut, 2	None
cGVHD	None	Limited, skin	None	None	None	Mild, gut	None	None
Transplant complications	HHV6 encephalopathy	None	AIHA	CMV, HHV6 reactivation	BK	Adeno, BK, and rhinovirus reactivation	HHV6 viremia, BK cystitis	HHV6 encephalopathy, AIHA, CMV reactivation
Survival status	Deceased	Alive	Deceased	Alive	Alive	Alive	Alive	Alive
Duration of follow-up (years)	1	10.5	2.6	3.3	7.3	10.9	2	4.3
Last follow-up clinical findings	Death due to progressive disease and septic shock 12 months post-UCBT; prior to death, seizures on AEDs	Nonambulatory, nonverbal, rare seizures on AEDs; receiving cerliponase alpha infusions (since 7 years post-UCBT)	Unexpected death 2.5 years post-UCBT; prior to death, seizures controlled on AEDs, loss of ambulation/language, part-time school, normal vision	Age-appropriate neurocognition, 6th grade and has an aide; no seizures, receives only visual support in age-appropriate school, uses Braille	Concerns for cognitive decline, in 9th grade, works at a bakery; seizures controlled on AEDs, receiving visual support, speech more slurred with stuttering	Rare seizures on AEDs, educational support for vision in age-appropriate school started his senior year, anxiety, legally blind, dysarthria	Talkative, 8th grade school for the blind, ambulates well with a walking stick for vision impairment	Seizures on AEDs, gestures for expression, decline in motor function so uses wheelchair, speaking less, suspected disease progression
Comparison of pre- and post-UCBT imaging studies, procedures, and Hamburg language-motor scores								
Pre-UCBT MRI	Cerebellar atrophy; T2 hyper intensity PVWM	Normal	Normal	T2 hyper intensity PVWM	Normal	Normal	Suspected mild b/L optic nerve atrophy	Mild T2 hyper intensity PVWM
Pre-UCBT EEG and treatment AED regimen if present	Abnormal: focal areas of neuronal dysfunction and epileptogenic potential; generalized slowing. AED(s): levetiracetam	Normal	Abnormal	Normal	Abnormal: area of epileptogenic potential	Abnormal: area of epileptogenic potential	Abnormal: interictal discharges. AED(s): levetiracetam	Normal
Pre-UCBT BAER	Abnormal	Abnormal	Abnormal	Normal	Normal	Normal	Normal	Normal
Pre-UCBT VEP	Normal	Normal	Normal	Abnormal	Normal	Abnormal	Normal	Normal
Last follow-Up MRI	Cerebellar atrophy; T2 hyper intensity, stable PVWM	Age advanced global brain parenchymal atrophy; ventricular system enlargement due to progressive central volume loss	Mild T2 hyper intensity of insular regions	Mildly age advanced parenchymal volume loss	Normal	Age advanced global parenchymal volume loss	Mild global parenchymal volume loss	Global atrophy; mild T2 hyper intensity PVWM
Last follow-up EEG and treatment AED regimen if present	Abnormal: epileptogenic potentials. AED(s): clonazepam, gabapentin, levetiracetam, valproic acid, phenytoin	Abnormal: mild-to-moderate intermittent generalized slowing, likely representing mild-to-moderate degree of encephalopathy. AED(s): clonazepam, levetiracetam	Abnormal: diffuse slowing. AED(s): clobazam, levetiracetam, zonisamide	Abnormal: epileptogenic potentials. AED(s): none	Abnormal: diffuse slowing/generalized brain dysfunction; interictal discharges consistent with multiple areas of epileptogenic potential. AED(s): levetiracetam	Abnormal: generalized background slowing; occasional bursts of high voltage delta waves with bitemporal predominance. AED(s): divalproex, levetiracetam	Abnormal: frequent bifrontal spike-wave discharges; left temporal focal slowing; diffuse background slowing. AED(s): none	Abnormal: diffuse slowing, possible sedation effect; multifocal spikes absent, but present prior. AED(s): levetriacetam
Last follow-up BAER	N/A	Abnormal	Abnormal	Normal	Normal	Normal	Abnormal	Abnormal
Last follow-up VEP	N/A	Abnormal	Normal	Abnormal	Abnormal	Abnormal	Normal	Abnormal
Hamburg Scale Pre-UCBT (M/L)	3/2	Unable to apply scale as patient was 3 months old	3/3	3/3	3/3	3/3	3/3	3/3
Hamburg Scale at last follow-up (M/L)	0/0 Prior to illness admission that lead to death	1/1	1/1	3/3	3/2	2/2	3/3	1/1

Abbreviations: AEDs, anti-epileptic drugs; BAER, brainstem auditory evoked response; BD, batten disease; EEG, electroencephalogram; MRI, magnetic resonance imaging; N/A, not assessed; PVWM, periventricular white matter; UCBT, umbilical cord blood transplantation; VEP, visual evoked potential.

^a^Indication that patient was enrolled and participating on the DUOC-1 protocol.

UCB grafts matched at a minimum of 4/6 human leukocyte antigen (HLA) loci and contained a median pre-cryopreservation total nucleated cell dose of 4.68 × 10^7^ cells/kg. Two patients (CLN3 = 1; CLN5 = 1) received double UCB transplants with a median total nucleated cell dose of 3.64 × 10^7^ cells/kg. All patients achieved and sustained full donor chimerism with neutrophil and platelet engraftment at a median time of 28.5 and 62 days post-UCBT, respectively.

Acute (a)GVHD manifestations included grade III skin (*n* = 1) and grade II skin/gut (*n* = 4). No severe chronic (c)GVHD was observed. Autoimmune hemolytic anemia occurred in 2 patients (CLN2 = 1; CLN5 = 1), resolving with rituximab and steroid treatments. Five experienced viral infections including HHV6 encephalopathy (*n* = 2) requiring foscarnet. No sinusoidal obstructive syndrome was observed. No adverse events related to DUOC-1 were reported among enrolled patients. Overall survival (OS) was 100% and 88% at 100 days and 24 months, respectively (95% CI, 33-97). No transplant-related mortality was observed.

### NCL clinical outcomes

#### CLN2

The median age at UCBT was 3 (0.25-4) years. One patient with CLN2 remains alive at last follow-up. This patient, asymptomatic at UCBT was 11 years old at last follow-up and has progressive MRI global atrophy, seizures, abnormal VEP and BAER, is nonverbal, has regression of milestones and receives cerliponase alpha intraventricular infusions. The other 2 patients were older at UCBT. One, symptomatic pre-UCBT, died of progressive disease 1-year post-UCBT. The other, initially asymptomatic, progressed post-UCBT and at last follow-up had seizures controlled on AEDs and loss of ambulation and language but participated in part-time school with an aide. This patient died unexpectedly while sleeping 2.5 years post-UCBT with no cause of death identified on autopsy. All 3 patients had significant regression depicted by post-UCBT HML scores of 0-1.

#### CLN3

The median age at UCBT was 8.5 (7-11.7) years. At 5.3 years median follow-up, all 4 CLN3 patients were alive. Each had early-stage disease (HML 3/3) at transplantation with vision loss but are now legally blind. Two remain neurocognitively appropriate, only requiring visual support in age-appropriate schools and maintain normal HML scores (3/3). While both of these had behavioral manifestations pre-UCBT, one had resolution of this issues post-UCBT without medication management and the other had notable improvement on 2 mood stabilizing agents. One patient pre-UCBT had no behavior concerns; however, developed anger issues within the first year post-UCBT that had improved at last follow-up while on medications. One patient had seizures controlled on AEDs (HML scores of 2/2), and remains in age-appropriate school with visual assistance. The fourth maintains normal motor function but developed cognitive decline with increasing speech difficulty (HML scores 3/2). While 1 patient had a normal pre-UCBT EEG, all 4 have abnormal EEGs post-UCBT. Three patients continue with normal BAERs.

#### CLN5

One patient, age 4.8 years at UCBT, is alive at 4.3 years follow-up post-UCBT. This patient, diagnosed while asymptomatic due to an older affected sibling, experienced notable regression from pre-UCBT HML scores of 3/3 to post-UCBT scores of 1/1.

## Discussion

Given the rarity of NCLs, diverse clinical presentations, and varied approaches/access to therapies, natural history and life expectancy are difficult to characterize. Therapeutic options are generally limited beyond supportive care and cerliponase alpha therapy for CLN2 patients, FDA-approved in 2017.^6^ HCT benefits in IMDs are via engrafted donor cells providing permanent enzyme replacement with the myeloid-derived microglial cell system, as the brain’s enzyme replacement vehicle.^6^ Hematopoietic cell transplantation (HCT), specifically UCBT has been shown to be effective in the treatment of several IMD with peroxisomal and lysosomal storage defects.^6,12-15^ Generally, in IMDs, HCT is better at preventing disease progression than in reversing already established disease manifestations, with early intervention associated with better outcomes.^15^ The use of sibling and/or related haplo-identical donors who are carriers of the disease may yield inferior results as compared to noncarrier donors.^6^ Despite promising results of HCT in IMDs, the tolerability and outcome of HCT for NCLs in pediatric patients remain undefined. Additionally, it is unknown if HCT will reduce the rate and tempo of disease progression and improve OS.

While our study is limited by sample size and the heterogeneity of NCLs, UCBT appears a safe and feasible therapeutic option. No patient in this cohort died from transplant-related complications, GVHD occurred but was treatable, graft rejection was not observed, and all patients had full donor chimerism at last assessment. Inferences regarding donor cell doses, risk of GvHD and other adverse events such as hemolytic anemia are limited by the small cohort and heterogeneity. Nonetheless, in this cohort, outcomes appeared poorer in those with advanced stage disease, rapidly progressing disease, and/or CLN2 diagnosis. Later disease onset, slower progressing disease, and/or CLN3 may correlate with disease stabilization although this may coincide with the natural history of CLN3 patients.^16^ Reports on cellular, gene, and enzyme therapies for the treatment of NCLs are emerging. Intravitreal enzyme gene therapy is emerging as progressive vision loss is not typically improved by enzyme replacement.^17^ Systemic disease modification will still be required with this adjunctive approach given the CNS pathologic contribution to functional vision.

In summary, UCBT appears safe and feasible in children with CLN2, CLN3, and CLN5. Prospective registries systematically characterizing the natural history of NCLs and capturing treatment-specific outcomes will improve our understanding of optimal therapeutic algorithms. Additional research trials are needed to determine the most effective treatment paradigm for less severe phenotypes. As new treatments become available, a sequential platform that includes UCBT with adjunctive local gene/enzyme-derived therapies is conceivable and future studies may help elucidate whether patients with NCLs are likely to benefit from UCBT.

## Data Availability

The data underlying this article will be shared on reasonable request to the corresponding author.

## Evidence before this study

There is no available curative therapy for patients with neuronal ceroid lipofuscinosis (NCL), a group of rare inherited neurodegenerative disorders that present in childhood and may lead to early death. Cellular therapy is at the forefront of exploration as a potential therapeutic option for these disorders and there is limited published literature regarding transplant outcomes. We searched PubMed using the search terms “Batten disease,” “neuronal ceroid lipofuscinoses,” “umbilical cord blood,” “bone marrow transplant,” “hematopoietic cell transplant,” “pediatric stem cell transplantation,” and “cellular therapy.” We sorted by clinical trial under article type and by most recent publication dates. Additionally, data presented in abstract form at major transplant meetings (American Society for Blood and Marrow Transplantation, EBMT, American Society of Hematology) were searched to find trials that had completed accrual and had reported data by the time of manuscript preparation. Searches were done from January 2023, until manuscript preparation on August 30, 2024.

## Added value of this study

With successful donor engraftment in all subjects and no observed transplant-related mortality, we show that UCBT is a safe and feasible potential therapeutic option for patients with NCL (as similarly performed in patients with other IMD).

## Clinical trial registration

UCB Transplant of Inherited Metabolic Diseases with Administration of Intrathecal UCB Derived Oligodendrocyte-Like Cells (DUOC-01); NCT02254863; https://clinicaltrials.gov/ct2/show/NCT02254863.
